# Prognostic implications for patients after myocardial infarction: an integrative literature review and in-depth interviews with patients and experts

**DOI:** 10.1186/s12872-022-02753-z

**Published:** 2022-08-02

**Authors:** Seon Young Hwang, Sun Hwa Kim, In Ae Uhm, Jeong-Hun Shin, Young-Hyo Lim

**Affiliations:** 1 School of Nursing, Hanyang University, Seoul, South Korea; 2 Department of Nursing, Hanyang University Medical Center, 222-1 Wangsimniro, Seondong-gu, Seoul, 04763 South Korea; 3 Division of Cardiology, Department of Internal Medicine, Hanyang University Guri Hospital, Hanyang University College of Medicine, Guri-si, Gyeonggi-do South Korea; 4 Division of Cardiology Department of Internal Medicine, College of Medicine, Hanyang University Medical Center, Seoul, South Korea

**Keywords:** Content analysis, Focus group, Heart failure, Myocardial infarction, Prognosis

## Abstract

**Background:**

As patients with myocardial infarction (MI) survive for a long time after acute treatment, it is necessary to pay attention to the prevention of poor prognosis such as heart failure (HF). To identify the influencing factors of adverse clinical outcomes through a review of prospective cohort studies of post-MI patients, and to draw prognostic implications through in-depth interviews with post-MI patients who progressed to HF and clinical experts.

**Methods:**

A mixed-method design was used that combined a scoping review of 21 prospective cohort studies, in-depth interviews with Korean post-MI patients with HF, and focus group interviews with cardiologists and nurses.

**Results:**

A literature review showed that old age, diabetes, high Killip class, low left ventricular ejection fraction, recurrent MI, comorbidity of chronic disease and current smoking, and low socioeconomic status were identified as influencing factors of poor prognosis. Through interviews with post-MI patients, these influencing factors identified in the literature as well as a lack of disease awareness and lack of self-care were confirmed. Experts emphasized the importance of maintaining a healthy lifestyle after acute treatment with the recognition that it is a chronic disease that must go together for a lifetime.

**Conclusion:**

This study confirmed the factors influencing poor prognosis after MI and the educational needs of post-MI patients with transition to HF. Healthcare providers should continue to monitor the risk group, which is expected to have a poor prognosis, along with education emphasizing the importance of self-care such as medication and lifestyle modification.

## Introduction

Myocardial infarction (MI) is a major cause of death worldwide and is continually on the rise consequent to aging and westernization of lifestyle. Many prospective cohort studies have been conducted on prognostic factors in post-MI patients, and the mortality rate increases as time elapses after the onset of MI. In a study of adults aged 65 and older in the United States, mortality rates were 51% at 5 years, respectively [1]. Another study in Taiwan, the cumulative incidence of major adverse cardiac events (MACE) increased by 5.9% and 13.8%, and mortality by 2.0% and 5.2% at 1 and 3 years, respectively [2]. MACE includes restenosis, stent thrombosis, MI recurrence, readmission due to heart failure (HF), coronary artery bypass surgery, and cardiac death [3]. In Korea, the number of patients diagnosed with MI increased by approximately 23.9% over a 5-year period [3]. The Korea Acute Myocardial Infarction Registry-National Institutes of Health (KAMIR-NIH) prospectively followed up 13,000 registered MI patients and reported MACE incidence and mortality rates of 9.6% and 4.3%, respectively [4].

MI has been reported to be a major cause of HF globally [5] and HF patients have been reported to have the highest rate of a history of MI (37.4%) [6], highlighting the need to pay attention to HF as an important complication of MI [7]. According to cohort studies, HF-related readmission rates in post-MI patients increased by 7.5% and 13.4% at 1 and 3 years, respectively [3], and a Swedish study also reported an increase of 11.4% at 1 year and 21.8% at 5 years [8]. Another study showed that, of the 1239 survivors with non-fatal MI, 29.1% developed HF during an average of 5.6 years of follow-up [9]. HF has been identified as the most potent factor that increases the prevalence and mortality in the later stages after MI onset [10]. Even with successful reperfusion and acute treatment in patients with MI, it is highly valuable to predict patients at high risk of HF or early clinical stages of HF as early as possible to provide therapeutic agents and interventions before left ventricular remodeling occurs [5].

In order for post-MI patients to live a healthy life without HF during long-term drug treatment, it is essential to maintain self-care such as risk factors and lifestyle modification along with medication compliance [5, 11]. Many nursing studies have been continuously conducted to determine the effects of educational interventions on lifestyle modification and self-care improvement, and factors affecting self-care for the prevention of secondary cardiovascular disease [11–13]. However, most of them were short-term studies of less than 1 year, and it is necessary to confirm the relationship between self-care and long-term prognosis.

Since many cohort studies with post-MI patients have been conducted to identify factors affecting MACE from a medical point of view, these longitudinal studies should be comprehensively reviewed to suggest risk groups that require intervention. In addition, in-depth interviews with post-MI patients who developed HF among MACE should confirm the relationship between their disease awareness, lifestyle, lack of self-care, and the need for education. Furthermore, through interviews with clinical experts, the justification of the nursing education direction that post-MI patients should modify their risk factors and maintain self-care behaviors based on disease awareness in order to prevent poor prognosis should be confirmed.

## Methods

### Study design

In order to identify poor prognostic factors after MI and to draw implications for the nursing direction for risk groups, a mixed design was used of reviewing the literature and interviewing patients with HF and clinical experts.

### Literature review on influencing factors of poor prognosis in post-MI patients

Scoping literature review was conducted in five steps according to the procedure [14], with the following as our study question: “What are the features of MI during long-term observation and the predictors of adverse outcomes of post-MI patients?” A literature search was performed in PubMed, Cumulative Index to Nursing and Allied Health Literature (CINAHL), Embase, and Web of Science. The dates were set at January 2010 until July 2020, and long-term cohort studies on MI patients that used clinical prognosis or health outcome as the dependent variable were included in the review. “acute MI and long-term clinical outcome” and “acute MI and long-term health outcome” were used as search terms, with “acute MI” and “acute myocardial infarction” being included in the search filter. The following were excluded from the review: (1) non-English, non-original articles, such as reviews, articles describing instrument development or experimental interventions, conference proceedings, and study protocols; (2) studies evaluating the efficacy of a drug; and (3) studies investigating the effects of a treatment or procedure.

**Fig. 1:**
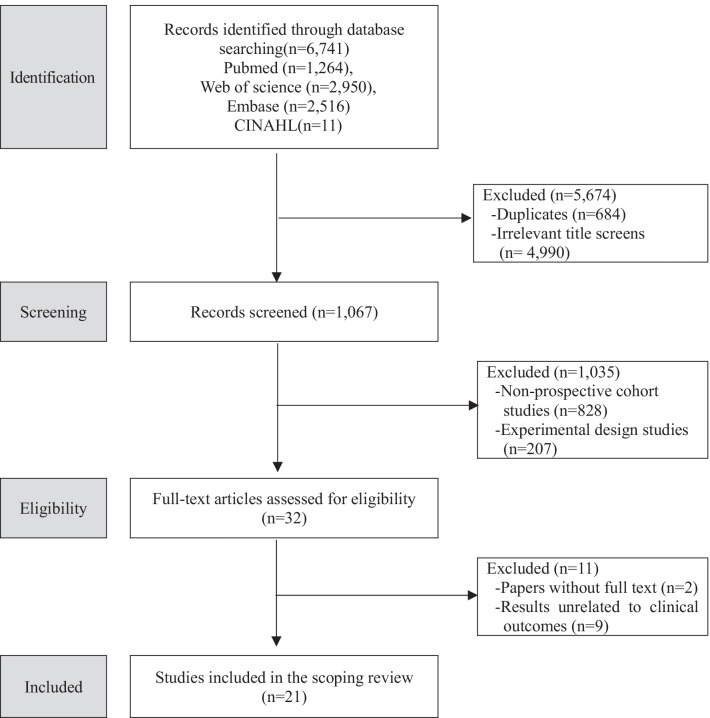
Flow chart of the literature selection process

A total of 6741 studies were identified via the search in electronic databases (PubMed, n = 1264; Web of Science, n = 2950; Embase, n = 2516; CINAHL, n = 11). After excluding duplicate searches (n = 684) and studies bearing titles that showed no relevance to the study topic (n = 4990), two researchers reviewed abstracts of 1067 studies in order to further exclude non-long-term studies (n = 828) as well as experimental studies, intervention studies, or systematic reviews (n = 207), resulting in a total of 32 selected studies. Among these 32 studies, those that did not provide full text (n = 2) or did not focus on or were irrelevant to clinical outcomes (n = 9) were excluded. Consequently, 21 studies in total were included in the final analysis (Fig. 1).

### In-depth interview with post-MI patients who progressed to HF

#### Participants and data collection

The participants were outpatients diagnosed with HF after more than 1 year had elapsed after undergoing percutaneous coronary intervention for MI at two medical institutions in Seoul, Korea (Table 1). The purpose of the study was explained to the interviewed patients and informed consent was obtained. We excluded patients with a left ventricular ejection fraction (LVEF) less than 40% at the time of first diagnosis of MI. The semi-structured interview questions were as follows: “What do you think about your illness?”, “What was your lifestyle like from MI onset until now?”, “What problems in your daily life do you think have brought upon this situation?”, “How do you intend to manage your illness?”, “What things do you wish your healthcare providers and family would manage for you?”. In-depth interviews with patients were conducted in a quiet place or interview room for 30–40 min at patients’ convenient time from October to December, 2020.

**Table 1:** Characteristics of interviewed post-MI patients with HF transition (n = 11)

Sex	Age	Education level	EF (%) at the time of MI	EF (%) at the time of HF	Period after MI (month)	Number of PCI	Comorbidity	Family CVD Hx^†^
M	54	Middle School	45.5	32.0	29	4	DM, CKD	No
M	81	University	63.7	44.9	19	1	HTN, DM	No
M	40	University	52.0	39.0	14	3		Yes
M	66	High School	48.0	49.0	17	2	HTN	No
M	72	Elementary	56.0	50.0	23	1	HTN, DM	No
M	67	High School	58.0	57.0	50	1	HTN	Yes
M	81	Middle School	57.0	41.0	72	2	DM Asthma	No
F	75	Elementary	65.0	42.0	68	2	HTN, DM, CKD	Yes
F	78	Elementary	64.5	20.0	173	4	HTN, DM, CKD	No
F	72	Elementary	48.0	35.0	74	2	DM, HTN	No
F	68	University	65.0	51.0	40	1	HTN Dyslipidemia	No

*MI* myocardial infraction, *HF* heart failure, *EF* ejection fraction, *PCI* percutaneous coronary intervention, *CVD* cardiovascular disease, *Hx* history, *M* male. *DM* diabetes mellitus, *CKD* chronic kidney disease, *HTN* hypertension, *F* female

^†^Family history of CVD includes diabetes, hypertension, dyslipidemia, arrhythmias, myocardial infarction, stroke, and heart failure

### Focus group interviews with clinical experts

#### Participants and data collection

Clinical experts for focus group interview (FGI) are 6 cardiologists with at least 10 years of clinical experience in the treatment of MI at university hospitals, and 6 nurses with averagely 10–15 years of clinical experience in cardiovascular nursing practice. The FGI was conducted for experts from February to March 2021, and was conducted after obtaining informed consent from them to participate in the study. As focus groups of no more than 10 participants facilitated active interviews [15], we constructed three focus groups of 3–4 participants per group and used the following semi-structured questions based on a literature review and research experience; “What are your thoughts on treating or caring for post-MI patients?”, “What are the difficulties you encounter as you treat or care the patients?”, “In your experience, what are the characteristics of post-MI patients with poor prognosis?”, “Who do you think are considered at high risk for MACE after MI?”, “What do you think is the most important patient factor when managing their condition after acute-phase treatment?” Each group interview was conducted for about 1 h in a conference room in the hospital, and was conducted and recorded by three researchers.

### Data analysis

For scoping review analysis, information on authors, country, subject, period, adverse clinical outcome, and influencing factors was extracted in summary format using an Excel data chart form. Three researchers performed intensive discussions and analysis during periodic meetings, and final agreements achieved through these meetings were recorded [14]. As scoping reviews provide comprehensive data on relevant research areas and do not require the integration of quantitative results to draw general conclusions, quality appraisals are not necessary [14]. Therefore, target studies were selected and summarized in compliance with the review scoping guidelines.

Qualitative analysis of interview data of patients and clinical experts was performed using the content analysis method [16]. First, two researchers transcribed the recorded interviews with patients and experts, and the principal investigator checked the accuracy. Second, in order to accurately grasp the contents of the interview, the transcript was read repeatedly, and meaningful sentences for each key question were underlined as a unit of analysis. Finally, three researchers extracted and classified meaningful statements and expressions to name sub-themes, and identified broader themes encompassing these sub-themes.

## Results

### Literature review on affecting factors of poor clinical prognosis in post-MI patients

Summary results of 21 cohort studies that identified factors affecting the clinical prognosis of post-MI patients are presented (Table 2). Although follow-up subjects in most studies included all age groups, one study included women aged 60–80 years [17] and four studies included patients with ST-elevated MI [18–21]. The follow-up duration after MI was approximately 1 year in 6 studies [21–26] with the longest follow-up period being 13 years [27], followed by 10 years [17, 28] and 8 years [20]. Adverse clinical outcomes as dependent variables were all-cause mortality [17, 19–24, 28–33], cardiovascular mortality [18, 28, 34], recurrent cardiovascular events or HF [2, 18, 21, 26, 27, 29], unplanned readmission [22, 25], and reduced health-related quality of life [35].

**Table 2:** Long term follow-up cohort study analysis and affecting factors in patients with MI

References	Source country	Subjects	Follow-up year	Adverse clinical outcomes (incidence rates)	Major affecting factors
Koren et al. [27]	Central Israel, 8 hospitals	1164 first MI patients	13	Recurrent MI or angina (45.6%)	Low education, low income, hypertension, diabetes, hypercholesterolemia, smoking, PCI, comorbidity index, Killip class, previous coronary heart disease
Kim et al. [22]	Korea (KAMIR-NIH, nationwide registry)	13,104 MI patients	1	Readmission and cardiac or cerebrovascular mortality (10.9%)	Age > 70, male, Killip class > 1, previous MI, previous angina, serum creatinine, PCI, PARADOCS (Pressure of ARtery elevAtion, Diabetes, Obesity, Cholesterol, Smoking) score
Norekvål et al. [17]	Norway 1 hospital	145 female MI patients(60-80y)	10	All-cause mortality (41%)	Old age, living alone, serum creatinine,, LVEF < 30%, marital status(divorced/widowed), low perceived health and quality of life
Daida et al. [30]	Japan (nationwide registry)	3597 ACS patients	2	All-cause mortality (6.3%)	Female, age ≥ 75, histories of MI, atrial fibrillation and cerebral infarction, hypertension, hyperlipidemia, smoking, eGFR < 60 ml/min, Killip class ≥ 2, peripheral arterial disease
Alhabib et al. [23]	Saudi Arabia (nationwide registry)	2233 ACS patients	1	All-cause mortality (8.1%)	Recurrent cardiac ischemia, recurrent MI, atrial fibrillation/flutter, previous stroke
Antoni et al. [18]	Netherlands 1 hospital	1453 STEMI patients	4	Cardiovascular mortality (4%) Hospitalization for HF (3%)	Age ≥ 70, Killip class ≥ 2, diabetes, left anterior descending coronary artery as the culprit vessel, multivessel disease, peak troponin T level ≥ 3.5 μg/L, LVEF ≤ 40%
Henderson et al. [28]	England and Scotland (National Statistics)	1810 NonST-ACS patients	10	All-cause mortality, (25.1%)@Cardiovascular death (15.1%)	Age, previous MI, heart failure, smoking status, diabetes, heart rate, and ST-segment depression
Steele et al. [19]	United Kingdom 1 hospital	3133 STEMI patients	3	Mortality (13.9%)	Old age, current smoker, ex-smoker, female, dyslipidemia, diabetes, previous MI, family history of chronic heart disease, chronic kidney disease stage IV or V, peripheral vascular disease
Barchielli et al. [20]	Italy (nationwide registry)	875 STEMI patients	8	All-cause mortality (49%)	Old age, Killip class > 1, cardiovascular or non-cardiovascular comorbidities, in-hospital cardiogenic shock, LVEF < 30%, treatment with aspirin and statin during hospitalization
Dohi et al. [33]	United States and Germany (multicenter registry)	8454 MI patients	2	Mortality (17.3%)@Recurrence of MI (3.3%)	Recurrent MI of unstable angina, diabetes, current smoker, multi-vessel disease, treatment of an in-stent re-stenotic lesion, low baseline hemoglobin and reduced creatinine clearance, antiplatelet agent factors, no use of statin at discharge
Chiang et al. [24]	Taiwan (multicenter registry)	3080 ACS patients	1	Mortality (22.4%)	Dual antiplatelet therapy ≥ 9 months, drug-eluting stents, chronic renal failure, in-hospital bleeding, NSTEMI, and antiplatelet discontinuation
Docherty et al. [36]	United Kingdom	13,202 MI patients	2	Sudden cardiac death (3.3%)	Old age, heart rate, smoking, Killip class, LVEF, history of prior atrial fibrillation, MI, HF, diabetes, eGFR
Pocock et al. [29]	Europe, America, Asia,Australia(Global registry in 25 countries)	9027 MI patients	3	Mortality (7.2%)@Recurrent cardiovascular events (1.4%)	Age ≥ 65 years, diabetes, second prior MI, chronic kidney disease, history of peripheral arterial disease or HF, cardiovascular hospitalization, diuretics, poor self-reported health
Munyombwe et al. [35]	England (nationwide registry)	9566 Survivors of MI patients	4	Reduced Health-related quality of life (69.1%)	Women, diabetes, previous MI and angina, chronic kidney disease, chronic obstructive pulmonary disease, cerebrovascular disease
Shah and Keeley [25]	United States	261 MI patients	1	Unplanned readmission (34%)	Recurrent MI, decompensated HF, low LVEF; diabetes
Carrick et al. [21]	United Kingdom	324 STEMI patients	1	All-cause mortality or HF(15%)	Hypertension, previous MI
Lopes et al. [31]	Global registry in 24 countries	14,703 MI patients	3	Mortality (2.2%)	Old age, baseline heart rate, creatinine clearance, new onset diabetes, previous MI
Gerber et al. [32]	Minnesota in United States	2596 MI patients	7.6	Mortality(42.9%)	Post-MI HF, MI severity, recurrent MI, comorbidity
Jernberg et al. [26]	Sweden	97,254 first MI patients	1	Cardiovascular events (18.3%)	Old age, prior MI, stroke, diabetes, HF, no index MI revascularization
Chen et al. [2]	Taiwan	11,183 Post MI patients	3	Cardiovascular events (13.8%)	Age, post-MI HF, hypertension, diabetes, prior stroke, chronic kidney disease, arterial fibrillation, underutilization of guideline-based medication
Park et al. [34]	Korea (KAMIR-NIH, nationwide registry)	10,455 MI patients	3.5	All-cause/cardiac death, MACE, HF (20.5%)	Old age(> 60), male, known/new onset diabetes, low BMI, low LVEF, multi-vessel disease, hypertension, dyslipidemia, prior stroke/angina/MI, renal failure

*ACS* acute coronary syndrome, *ACEi* angiotensin converting enzyme inhibitor, *STEMI* ST-segment elevation myocardial infarction, *NSTEMI* non ST-segment elevation myocardial infarction, *MI* myocardial infarction, *ARB* angiotensin receptor blocker, *eGFR* estimated glomerular filtration rate, *HF* heart failure, *KAMIR-NIH* Korea acute myocardial infarction registry-national institute of health, *PCI* percutaneous coronary intervention, *TIMI* thrombolysis in myocardial infarction. *LVEF* left ventricular ejection fraction, *MACE* major adverse cardiovascular events

Older age [2, 17, 18, 20, 22, 26, 28–31, 34, 36] and the recurrence of MI or angina [18, 22–28, 32–36] were the most frequent factors affecting the adverse outcomes of post-MI patients, followed by diabetes prevalence [2, 18, 19, 22, 25–29, 31, 33, 35, 36], high Killip class [18, 20, 22, 27, 31, 36] and LVEF [17, 18, 20, 25, 31]. As prevalent diseases, hypertension [2, 21, 27, 30, 32], chronic kidney disease [2, 24, 29, 33, 35], prior stroke [19, 30, 35] and HF [25, 26, 28, 29], atrial fibrillation [2, 25, 35] and multi-vessel involvement during infarction [19, 36] were also found to be affecting factors. Smoking as a lifestyle factor was identified as a factor that negatively affects the prognosis in several studies [19, 22, 27, 28, 30, 33, 36]. As demographic factors, poor socioeconomic status such as low education level [17, 27] and low income [27], marital status (divorced or widowed) [17], living alone [27] and poor self-reported quality of life [17] or health [29] were identified.

### Disease perception and self-care experience in post-MI patients who progressed to HF

After analyzing the perception of the disease and self-care experience through an in-depth interview with the patients, 6 subthemes emerged and were subsequently grouped into 3 broader themes that encompassed them (Table 3).

**Table 3:** Disease perception extracted from in-depth interviews with post-MI patients

Themes	Sub-themes	Statements
Exhaustion from endless treatment	Initially shocked but soon became oblivious the disease	*“It was a bit shocking at first. I had an ordinary life just like everyone else, but wow, things that I only saw on TV do really happen to me all of a sudden…. But now, some time has passed, and I’ve become somewhat oblivious…”*
	Getting tired from repeated hospitalizations due to recurrence	*“I took my meds and went to my hospital appointments just as told by my doctor, but this disease I guess is hard to cure. Recently, I just watch TV all day without anything else to do and am depressed. I’m in so much pain even when I do everything.*”
Lack of understanding about the disease	Inadequate self-care despite long term progression	*“I thought all I have is to quit smoking for AMI…. They tell me to eat my food with less salt, but that’s hard. What’s most important is to take my meds but I forget that often….”*
	Becoming passive in disease management	*“They told me I need to control my diabetes well so I think I take my prescriptions well but I don’t know why it’s not controlled well. I just eat whatever I want because I’m taking meds. I don’t check my blood sugar.”*
Desperately seeking help from healthcare providers	Difficulty in approaching busy healthcare providers	*“Even if I wanted to ask about something, I forget about all that when I meet my doctor. Everyone’s so busy and there are so many patients waiting. I just see the doctor for a few minutes during my appointment, so I just get my medications and come home….”*
	Desire for continuous attention and management from healthcare providers	*“I think I’m doing well but I don’t know if I’m really doing well because no one monitors me whether I’m doing well or not…”*

*MI* myocardial infarction

- Theme 1. Exhaustion of endless treatment

Initially shocked but soon became oblivious to the disease: The patients were initially shocked by the diagnosis of MI, but forgot the severity of the disease as the symptoms such as chest pain and shortness of breath disappeared and resumed daily activities.

It was a bit shocking at first. I had an ordinary life just like everyone else, but wow, things that I only saw on TV do really happen to me all of a sudden…. But now, some time has passed, and I’ve become somewhat oblivious...But it’s still scary.

Getting tired from repeated hospitalizations due to recurrence: Majority of patients expressed burden with increasing cost, felt sorry to their family, and became tired from repeated readmissions and procedures due to recurrent heart disease during treatment.

I have to take medications every day so I get frustrated. I underwent stenting a couple times already. I feel sorry to my family. I don’t want to do anything because it doesn’t seem like I’m improving.

- Theme 2. Lack of understanding about the disease

Inadequate self-care despite long term progression: Patients had a poor understanding about how to manage their disease or had no idea what to specifically do. Some patients expressed that even when they have retrieved information, they are clueless as to how to apply the obtained information to themselves.

I thought all I have is to quit smoking for treatement…. They tell me to eat my food with less salt, but that’s hard. What’s most important is to take my meds but I forget that often. I don’t know how long I have to keep taking medications.

Becoming passive in disease management: While suffering from their condition for a long period, the patients frequently missed their medications, felt annoyed about having to modify their lifestyle, and became passive in disease management. Patients did not have an accurate understanding about MI and thought that they only needed to correctly take the prescribed medications.

They told me I need to control my diabetes well so I think I take my prescriptions well but I don’t know why it’s not controlled well. I just eat whatever I want because I’m taking meds. I don’t check my blood sugar.

- Theme 3. Desperately seeking help from healthcare providers

Difficulty in approaching busy healthcare providers: The patients expressed that asking about matters that they had in mind or about their treatment was difficult even when they met with their healthcare providers during their appointments because the they seemed busy with all waiting patients.

Even if I wanted to ask about something, I forget about all that when I meet my doctor. Everyone’s so busy and there are so many patients waiting. I just see the doctor for a few minutes during my appointment, so I just get my medications and come home.

Desire for continuous attention and management from healthcare providers: Most patients desired that their healthcare providers pay close attention to them and take care of them. Additionally, they wanted healthcare providers to monitor whether their self-care is sufficient and whether they are adequately managing their condition.

I’m worried about how I should live from now on. I wish the hospital staff would take care of me better. But I forget on my own and even when I decide to do well, it’s hard to maintain….

### Experts’ perspectives on factors affecting the poor prognosis of post-MI patients

FGIs conducted on a panel of healthcare providers (physicians, nurses) who treated and provided care to MI patients resulted in 11 subthemes, and these subthemes were grouped into 4 themes (Table 4).

**Table 4:** Perspectives of physicians and nurses on the prognosis of post-MI patients

Themes	Sub-themes	Statements
Patients and situational factors in the acute phase increase the risk of poor prognosis	Irreversible acute-phase situational factors	*“Failure to manage diabetes and continuation of smoking exacerbate the lesions and increase the chance for a second procedure.” (Physician)*
	Patient's underlying chronic disease	*“The readmission rate is higher in individuals with uncontrollable diabetes and hypertension. Patients experiencing frequent relapses often progress to HF.” (Nurse)*
Self-awareness as a chronic condition that must go together for a lifetime needed	Entering a new disease management	*“In many cases, patients falsely believe that their illness is completely cured after the procedure.” (Physician)*
	Recognition that the disease can recur at any time	*“It’s imperative that patients are aware that it can relapse at any time if they do not engage in self-care after the procedure and that they must manage the condition throughout their lives.” (Nurse)*
Importance of maintaining healthy behavior after the acute phase	Difficulty in self-care compliance	*“Medication adherence, regulation of risk factors such as smoking cessation, and ensuring that patients don’t miss their hospital appointments determine their prognosis. Nevertheless, these are really difficult for patients to comply with.” (Physician)*
	Meaning of first discharge education from hospital	*“I believe that properly educating patients after an acute-phase procedure before they are discharged determines their first year.” (Physician)*
Strategies and educational efforts are needed for lifelong self-care of high risk patient	Tailored education on risk factors of patients for behavioral change	*“Most physicians do educate patients somewhat. However, we need to ensure that patients understand and comply with it with adequate education using learning materials, but we actually don’t have much time for that.” (Physician)*
	Importance of cardiovascular nurses for continuous monitoring	*“Even if the patient does not see the doctor often, it can be changed positively if the outpatient nurse keeps track of progress and monitors changes in condition along with training on lifestyle modification.” (Nurse)*

*MI* myocardial infarction

- Theme 1. Patients and situational factors in the acute phase increase the risk of poor prognosis Uncorrectable acute-phase situational factors and the patient's underlying chronic disease influence prognosis: Physicians and nurses said the timing of coronary ischemia, the time of arrival at the hospital, and the patient's health status, with or without appropriate treatment, are unavoidable factors that determine the outcome and prognosis of treatment. They also said that older post-MI patients have chronic conditions such as diabetes or high blood pressure, and if these conditions are not effectively controlled, relapses can lead to repeated hospitalizations, which can eventually lead to HF.

In the acute phase, how quickly the patient comes to the hospital and what vessels are involved are extremely critical.”, “The readmission rate is higher in individuals with uncontrollable diabetes and hypertension. Patients experiencing frequent relapses often progress to HF.

- Theme 2. Self-awareness as a chronic condition that must go together for a lifetime needed

Recognition of entering a new disease management: Physicians and nurses believed that MI is an entry to a new disease that must be managed with medications throughout one’s lifetime. In particular, it is important for patients to have self-awareness that it is a disease that requires lifelong treatment rather than ending with acute treatment.

With advances in medical technology, patients stay in the hospital for a shorter period of time. This makes them think that this is a manageable and their treatment is now over, but it’s important to instill that heart diseases must be managed as chronic conditions.” (Physician)

- Theme 3. Importance of maintaining healthy behavior after acute phase

Difficulty in self-care compliance: They thought that the degree to which patients modify their lifestyle after treatment beyond simply not missing any medications and hospital appointments is important

Compliance with medication and lifestyle management such as smoking cessation has an impact from 1 year after the acute phase. (Physician)

Meaning of first discharge education from hospital: They thought that the discharge education provided to patients after MI treatment is crucial and that the patients’ acquisition of a correct perception about their disease and the implementation of necessary measures in their lifestyle during this period are important predictors of long-term prognosis.

I believe that properly educating patients after an acute-phase procedure before they are discharged determines their first year. (Physician)

- Theme 4. Strategies and educational efforts are needed for lifelong self-care of high risk patients

Physicians said that all they had to do was check the symptoms and prescribe medication because the waiting list was long and the treatment time was short due to the unrealistic medical bills in Korea. So, it is impossible to monitor the patient's medication compliance or lifestyle. However, both nurses and doctors agreed that the following strategic efforts based on close interaction with patients are needed to improve the patient's prognosis.

Tailored education on risk factors of patients for behavioral change: Physicians and nurses agreed that an education and counseling through which they can provide patients with immediate feedback and promote active patient participation is necessary.

People have different risk factors. Thus, it’s important that when providing individual education, the patient’s risk factors must be identified first and emphasized in the education.” “An environment (time, space, medical fee) where healthcare providers can provide adequate education needs to be fostered. (Nurse)

Importance of cardiovascular nurses for continuous monitoring: Nurses and physicians agreed that in-hospital professional cardiovascular care nurse intervention is essential to help improve medication and lifestyle modification in post-MI patients, particularly those with poor prognostic factors.

Due to the short time with the physician in outpatient care, patients have a hard time asking questions that they had. If an outpatient cardiovascular nurse is designated, they can meet with patients before and after they meet with the doctor, and it would have a positive impact on patients’ prognosis. (Nurse)

## Discussion

As a result of literature review, old age and recurrence of MI or angina pectoris were the significant factors affecting poor prognosis, such as mortality, in post-MI patients. The findings that old age is a major factor in determining the long-term prognosis of post-MI patients were found in not only registration studies conducted in many foreign countries [29, 30, 36], but also the KAMIR-NIH studies of patients with MI in Korea with the participation of 36 tertiary medical institutions [22, 34]. Although aging is an uncontrollable risk factor and the largest contributor to cardiovascular disease [37], it suggests that more efforts are needed to prevent HF in elderly post-MI patients. A total of 14 studies identified type 2 diabetes as a predictor of poor prognosis, making it the second most potent factor. This result is supported by the findings of multicenter studies involving patients with diabetes, which reported that participants engaged in strict blood glucose control exhibited a 57% reduced risk of MACE, including MI, stroke and cardiovascular death from at 17 years later [38] and had a 33% lower all-cause mortality rate over 27 years [39]. Another cohort study that followed up patients with diabetes for 2.9 years reported that MI recurrence and mortality rates were 1.74 and 2.43 times higher in the diabetic MI group, respectively [40], suggesting that strict blood glucose control is essential during self-care among post-MI diabetic patients.

The recurrence of MI was identified as a significant predictor of increased mortality in a 7.6 year [32] and 10-year [27, 28], and 3-year global registry cohort studies including Europe and Asia [29] respectively. Chronic kidney injury has also been shown to influence long-term prognosis in several literature reviews [2, 24, 29, 33, 35]. In patients with ST-segment elevation MI, kidney injury due to increased serum creatinine in the acute phase was found to be an independent predictor of long-term mortality [41], so continuous monitoring is necessary even for patients with early kidney injury. Therefore, the elderly, diabetic patients, patients with recurrent MI and kidney injury should be considered as high risk groups for poor prognosis, such as HF transition and death. For these high-risk patients, it is necessary to provide customized risk factor education and continuous observation before discharge or during outpatient visits.

In addition, a high Killip classification at the first diagnosis of MI and a low LVEF value, an indicator of left ventricular systolic function, were confirmed as prognostic factors for all-cause death or cardiac death in cohort studies conducted for 2–10 years [17, 18, 30, 36]. More special follow-up is needed for patients with abnormalities in these medical indicators. Only a few studies have reported that smoking as an individual's lifestyle [19, 30, 36], low socioeconomic status [27], living alone, low quality of life [17], and poor self-reported health [29]. More research is needed to determine whether lifestyle modifications including smoking cessation, diet, and exercise in post-MI patients affect the improvement of adverse prognostic indicators in post-MI patients over a long-term course.

Three themes emerged in the in-depth interviews with post-MI patients who developed HF. The patients experienced ‘exhaustion from endless treatment’ but became oblivious to their disease, which initially had been a shocking news. In particular, they had a ‘lack of understanding about the disease.’ Despite suffering from the disease for a long time, they had little knowledge about the disease, engaged in inappropriate self-care, and remained passive in disease management. These results support that low or negative disease perception among post-MI patients affects their quality of life and may lead to anxiety or depression [42], forcing them to adopt avoidance as a coping mechanism and thereby affecting their health in a vicious cycle [43]. Furthermore, fatigue increases with age in post-MI patients due to their low physical activity level [44]. Consequently, patients tend to become more passive in disease management. Thus, high-risk patients require individualized nursing care and should be screened with consideration of their individual and social characteristics from the day of their discharge after MI treatment. Finally, the patients were ‘desperately seeking help from healthcare providers,’ expressed the desire for healthcare providers to provide the necessary assistance and to stay with them, and experienced difficulty in approaching them who seemed to be busy all the time. A qualitative study reported that external motivation, by spouses or healthcare providers is important to encourage MI patients and that individual preferences should be taken into consideration [45]. Through interviews with post-MI patients who progressed to HF, it was found that risk factors identified in the literature exist, and that awareness of the disease and self-care are insufficient. Recently, it has been reported that through wearable devices such as smartphone apps, patients can help improve their lifestyle by inputting their own diet, blood sugar, weight, walking activity, etc., and it is reported that these activities significantly lower the risk [46, 47]. Therefore, it is necessary for cardiovascular nurses to provide motivation for taking medication and improving lifestyle by using these apps along with counseling education for high-risk post-MI patients.

In the present study, FGIs were conducted on physicians and nurses who treated and provided care to post-MI patients, and 7 prognosis-related subthemes emerged, which were subsequently grouped into 4 overarching themes. The most potent factor of prognosis in post-MI patients was irreversible patient and situational factors such as the delay before hospital arrival, location of necrosis, and existing chronic condition. This supports the results of several quantitative studies reporting on the influence of patients’ situational factors or other variables [48, 49]. In particular, diabetes increased the risk of progression to HF after MI by 1.58 times [34], MI recurrence by 1.76 times, and all-cause mortality by 1.90 times [40], suggesting that the provision of more systematic individual and family education and continuous monitoring of post-MI patients with diabetes at the time of onset are necessary.

Physicians and nurses considered it important for MI patients to realize that discharge after acute treatment is not the end but the beginning of a new disease, that is, 'self-awareness as a chronic condition that must go together for a lifetime needed'. Previous studies reported that patients’ awareness of their disease influences their self-efficacy and compliance with self-care [12, 50]. Additionally, an accurate perception of “what to do” can result in positive improvements, underscoring the necessity to examine the awareness level of patients in clinical practice and devise strategies for tailored education. Another essential theme was ‘importance of maintaining healthy behavior after the acute phase.’ Both physicians and nurses emphasized the importance of first pre-discharge education after admission. Previous studies reported the effectiveness of pre-discharge education [51, 52] and showed that pre-discharge education increased the awareness, compliance with self-care, knowledge, and self-efficacy of patients with cardiovascular diseases [13]. Finally, physicians and nurses thought that ‘strategy and educational efforts are needed for lifelong self-care of high risk patient’ to prevent side effects during the treatment process. The physicians thought that there was not enough time for consultation other than taking medicine or checking symptoms due to the short meeting time at the outpatient clinic, so professional personnel were needed to provide long-term continuous education. The nurses also fully agreed with this opinion, and said that based on the close interaction between patients, nurses, and doctors, cardiovascular nurses would be able to monitor risk factors and provide counseling for behavior change. Numerous nursing studies have been conducted on educational interventions and positive effects for myocardial infarction patients [53, 54]. Unlike the subjects of this study, in a qualitative study of 22 patients who maintained good self- care for an average of 10 years or more after MI, it was suggested that the family and healthcare providers need to strengthen support based on the patient's preference and autonomy [45]. This suggests that patients' non-compliance after myocardial infarction should be recognized as a holistic problem and that health care providers should establish a customized treatment plan in cooperation with patients. However, the characteristics of the subjects of Hanna et al.’s study [45], such as age and underlying disease, may be different from those of this study, so a qualitative or quantitative study comparing the healthy group after MI and the HF transition group is needed in the future.

This study is meaningful in that it was investigated through a mixed research method in order to prepare a nursing strategy necessary for the prevention of complications such as heart failure for a long period of time after acute stage treatment in the increasing number of post-MI patients. In addition, by interviewing post-MI patients who transitioned to HF, it was confirmed that they lacked awareness and self-care as chronic disease patients. In outpatient-based practice, it has the strength to draw the implications of physicians and nurses for the need for educational interventions by cardiovascular nurses for poor prognosis risk groups.

However, this study has some limitations. First, some relevant studies may have been excluded from literature review because they did not use search terms. Second, the literature review was conducted on all MACEs, such as readmission, death, and transition to HF in post-MI patients, and the focus group interview was conducted only with patients who had undergone readmission due to transition to HF. Therefore, there is a limitation in generalizing the results of the study to the case of all patients with MACE. Finally, the sample size of in-depth interviews with patients and doctors and nurses was not large, and these interviews were conducted in a single university hospital, limiting the generalization of the results.

## Conclusion

Through literature review, old age, diabetes, recurrent MI, Killip class and LVEF at first diagnosis, smoking, and low socioeconomic status were identified as poor prognostic factors such as mortality in post-MI patients. In-depth interviews with post-MI patients who transitioned to HF with these influencing factors confirmed that they had low awareness of chronic diseases, neglected self-care, and wanted attention and help from health providers. Clinical experts agreed that self-care such as adherence to medication, maintenance of a healthy lifestyle, and thorough management of comorbidities, such as diabetes, is essential with the recognition that a new disease enters after acute treatment. To this end, it was emphasized that consultation and education based on individualized interaction by a cardiovascular nurse for high-risk patients were needed as an outpatient basis.

## Data Availability

The datasets generated and/or analyzed during the current study are available from the corresponding author upon reasonable request.

## Abbreviations

MI: Myocardial infraction
MACE: Major adverse cardiac events
KAMIR-NIH: Korea Acute Myocardial Infarction Registry-National Institutes of Health
HF: Heart failure
CINAHL: Cumulative Index to Nursing and Allied Health Literature
LVEF: Left ventricular ejection fraction
FGI: Focus group interview
